# Access to childbirth care services in the interior of Pernambuco, Northeast region of Brazil

**DOI:** 10.11606/s1518-8787.2023057004335

**Published:** 2023-03-15

**Authors:** Régia Maria Batista Leite, Thália Velho Barreto de Araújo, Maria Rejane Ferreira da Silva, Antônio da Cruz Gouveia Mendes, Maria do Socorro Veloso de Albuquerque

**Affiliations:** I Fundação Oswaldo Cruz Instituto Aggeu Magalhães Programa de Pós-Graduação em Saúde Pública Recife PE Brasil Fundação Oswaldo Cruz. Instituto Aggeu Magalhães. Programa de Pós-Graduação em Saúde Pública. Recife, PE, Brasil; II Universidade Federal de Pernambuco Centro de Ciências Médicas Programa de Pós-Graduação em Saúde Coletiva Recife PE Brasil Universidade Federal de Pernambuco. Centro de Ciências Médicas. Programa de Pós-Graduação em Saúde Coletiva. Recife, PE, Brasil; III Universidade de Pernambuco Faculdade de Enfermagem Nossa Senhora das Graças Recife PE Brasil Universidade de Pernambuco. Faculdade de Enfermagem Nossa Senhora das Graças. Recife, PE, Brasil; IV Fundação Oswaldo Cruz Instituto Aggeu Magalhães Departamento de Saúde Pública Recife PE Brasil Fundação Oswaldo Cruz. Instituto Aggeu Magalhães. Departamento de Saúde Pública. Recife, PE, Brasil

**Keywords:** Maternal and Child Healthcare Services, provision & distribution, Quality, Access, and Evaluation of Healthcare, Barriers to Access to Healthcare, Ecological Studies

## Abstract

**OBJECTIVE:**

To analyze the access of women to the public health system network to childbirth care, highlighting the barriers related to the “availability and accommodation” dimension in a health macroregion of Pernambuco.

**METHODS:**

Ecological study, conducted based on hospital birth records from the Hospital Information System of the Brazilian Unified Health System (SUS), and information from the state’s Hospital Beds Regulation Center, about women residing in health macroregion II, in 2018. Displacements were reviewed considering the geographic distance between the municipality of residence and that of the childbirth; estimated time of displacement of pregnant women; ratio of shifts blocked for admission of pregnant women for delivery; and the reason for unavailability.

**RESULTS:**

In 2018, health macroregion II performed 84% of usual risk childbirths, and 46.9% of high-risk childbirths. The remaining high-risk childbirths (51.1%) occurred in macroregion I, especially in Recife. The reference maternity for high-risk childbirths in that macroregion had 30.4% of the days of day shifts and 38.9% of the night shifts blocked for admission of childbirths; the main reason was the difficulty in maintaining the full team in service.

**CONCLUSIONS:**

Women residing in the health macroregion II of Pernambuco face great barriers of access in search of hospital care for childbirth, traveling great distances even when pregnant women of usual risk, leading to pilgrimage in search of this care. There is difficulty regarding availability and accommodation in high-risk services and obstetric emergencies, with shortage of physical and human resources. The obstetric care network in macroregion II of Pernambuco is not structured to ensure equitable access to care for pregnant women at the time of childbirth. This highlights the need for restructuring this healthcare services pursuant to what is recommended by the *Cegonha *Network.

## INTRODUCTION

In the last three decades, Brazil has shown advances in women’s healthcare as a result of efforts and initiatives by the government and society, especially the organized women’s movement. However, high levels of preventable maternal, fetal, and neonatal morbidity and mortality still persist^1^.

This persistent situation justified the implementation of several programs, policies, and health strategies, including the *Rede Cegonha* (RC). The RC was launched in 2011 as a strategy for organizing maternal and child care, aiming to change the model of delivery and birth care and reduce maternal and neonatal morbidity and mortality^2^.

This shift in the obstetric and neonatal care model was discussed by managers in various health regions of the country, including the adhesion of the health macroregion II of Pernambuco^3^, entering commitments to change childbirth and delivery care practices^4,5^. This networked organization is important to ensure women’s access to healthcare services, especially those for childbirth care, avoiding the occurrence of disorderly flows of pregnant women, which can lead to pilgrimage of women seeking care for childbirth^6^. An important aspect to overcome access difficulties is the organization of this network to overpower the persistence of great differences in the distribution and quality of services offered between regions and health macroregions^7^.

However, it is known that despite the many efforts toward advancing actions and services in health regions, we agree with Shimizu et al.^8^ when they say that access to healthcare services is difficult, especially to those of greater complexity, specialized services, and hospitals with more technological resources. This demands users to travel long distances to get the necessary care.

In this sense, it is understood that access to healthcare services is an important component in all health systems. However, there is no consensus in the literature about its concept^9,10^, varying among authors and changing over time^11^. For the purpose of this article, we adopted the concept of access systematized by Levesque et al.^12^, which defines it as the opportunity to reach and obtain adequate healthcare services in situations of perceived need for care. It is results from the interface between the characteristics of individuals, families, social and physical environments and the characteristics of health systems, organizations, and providers.

In their theoretical model, the authors formulated five dimensions involving the concept of access (accessibility, acceptability, availability and accommodation, affordability, and appropriateness) and five users’ individual abilities (ability to perceive; to seek; to reach; to pay; to engage). In this paper we chose to work with the “availability and accommodation” dimension, which encompasses the physical existence and production potential of the service, involving structural aspects, facilities, distribution, human resources, contexts, and urban geography. It is configured in the fact of reaching a healthcare service in a timely manner^12^.

In this sense, an important point is to review the access of women served in the public network to childbirth care services in the health macroregion II of Pernambuco, highlighting the barriers related to the dimension “availability and accommodation”.

## METHODS

A population-based ecological study, conducted based on hospital birth records from the the Hospital Information System (*Sistema de Informação Hospitalar) of the Brazilian Unifed Health System *(SIH-SUS), and information from the state’s Hospital Beds Regulation Center (*Central de Regulação de Leitos*) about women residing in health macroregion II, from January 1 to December 31, 2018.

In Pernambuco, the network organization is ruled in the Regionalization Master Plan (*Plano Diretor de Regionalização*, PDR), with the redefinition of the territorial organization of the state in 12 health regions grouped into four health macroregions, aiming to subsidize the organization of the health network in a regionalized, hierarchical, and resolutive manner^3^. Health macroregion II is located in the *Agreste* region of the State of Pernambuco. It is composed of health regions IV and V. The IV health region has its administrative headquarters in Caruaru and comprises 32 municipalities, with a population of 1,324,382 inhabitants^3^ (415,471 women of childbearing age). It houses a maternity that is reference to the macroregion II regarding high-risk pregnant women, but it lacks Intensive Care Unit (ICU) for these women. The health region V, with administrative headquarters in the municipality of Garanhuns, comprises 21 municipalities and 534,793 inhabitants^3^ (163,667 women of childbearing age) and has a regional maternity hospital for high-risk pregnant women.

To analyze access through the dimension “availability and accommodation” proposed by Levesque et al.^12^, we used the displacement of women through the following variables: distance traveled by women seeking assistance for delivery of usual risk and high obstetric risk, and travel time between the municipality of residence and of occurrence. The variables related to the care delivered to high obstetric risk childbirths, reference to women living in macroregion II. To that, the following variables were analyzed: proportion of days of day- and night- shifts closed for admission of pregnant women at the time of delivery and the reason for the closing of shifts, based on information from the Hospital Bed Regulation Center of Pernambuco. This Center works with the support of a call center that receives calls from all health units in the state that deal with urgency/emergency cases, ICU and obstetrics. The center consolidates information about demands and services provided.

The inter-municipal distances were submitted to geoprocessing based on calculations of the distances between the centroids of the municipalities of residence and those of occurrence. When calculating the geographic displacement, intra-municipal displacements were not considered; therefore, if the delivery occurred in the municipality of residence, the distance was equal to zero, according to the methodology proposed by Almeida et al^13^.

Geographic distances and travel time were estimated and grouped into five categories (< 30km/30min; ≥ 30 - < 60km/30 to 60min; ≥ 60 - < 90km; ≥ 90km < 120km/90 to 120min; and < 120km/above 120 min), including all municipalities in macroregion II, taking into account pregnant women at the time for habitual and high-risk deliveries, according to the definitions of the Brazilian Ministry of Health incorporated by the SIH. The SIH database and information on the operation of the shifts were provided by the Pernambuco State Health Department.

To review the displacement of pregnant women, flow maps were built using the ArcGIS 10.4 program, differentiated for pregnant women with usual risk deliveries and for pregnant women with high-risk deliveries, having as unit of analysis the health regions and health macroregion.

The flow maps represent the displacement of women in space, demonstrating the movement by means of vectors drawn to represent the itinerary traveled by the pregnant women, characterized by thicknesses proportional to the number of childbirths. Flows were categorized for pregnant women with usual risk deliveries: IV health region (11-50 deliveries; 51-100 deliveries; and ≥ 101 deliveries) and for V health region (11-50; 51-100; and ≥ 101 deliveries). As for deliveries of high-risk pregnant women, flows were defined in the same way for both health regions: 1-10 deliveries; 11-50 deliveries; 51-100 deliveries; and ≥ 101 deliveries. In this sense, the dominant flow was defined as the largest flow (starting from 10 deliveries) in each municipality, which allowed identifying the framework of the connections network^14^.

The study of flows is a key component for observing issues related to access, identifying the distances traveled^14^, helping to identify the organization of services in a regionalized network. Similarly, the organization of the service for high-risk obstetric delivery is also an important component of “availability and accommodation” to find to what extent the service is structured in the health macroregion to reduce barriers to access.

The research was approved by the Research Ethics Committee of the Research Center Aggeu Magalhães/Fiocruz, according to Resolution 510/16, under CAAE n. 63796717.4.0000.5190.

## RESULTS

**Figure 1:**
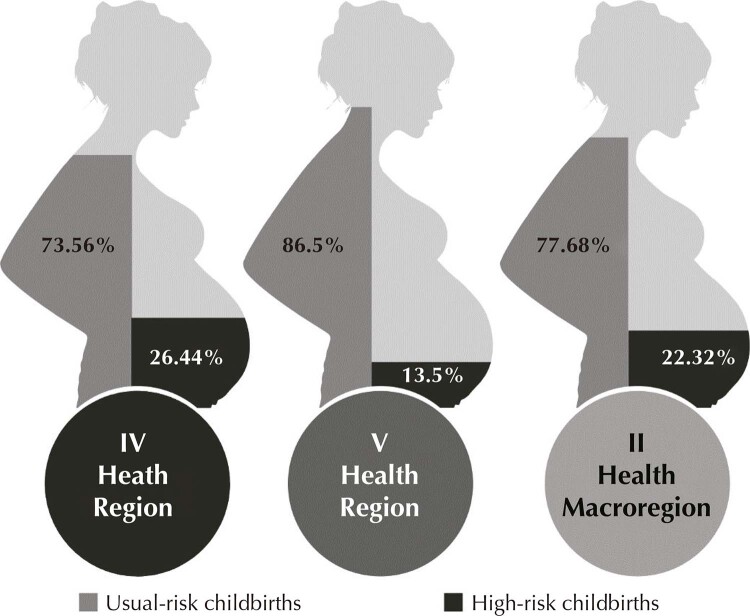
Proportion of usual-risk and high-risk childbirths according to health region of residence and health macroregion II, 2018.

Source: SIH/Datasus, 2018.

In 2018, 77.7% of childbirths occurred in health macroregion II were classified as usual risk and 22.3% as high-risk. When considering the health regions, it was found that 26.4% of childbirths from high-risk pregnant women were from residents in the IV health region, while in the V region this figure was 13.5% (Figure 1).

Regarding the place of delivery, it was observed that 84% of childbirths from high-risk pregnant women were in the health macroregion II. However, when analyzing the childbirths from women with usual and high obstetric risk occurring outside their region of residence, it is found that 66.4% of deliveries from pregnant women with usual risk occurred in municipalities up to 30 km away, and 17.1% occurred in municipalities more than 120 km away. Regarding childbirths from high-risk pregnant women, 49.1% of the women needed to travel more than 120 km, taking more than two hours of travel to have their childbirth or obstetric emergency attended. These results show that many women had to travel to very distant municipalities to get assistance, even though they were high-risk pregnant women (Table 1).

**Table 1:** Distribution of deliveries with usual and high obstetric risk, according to geographic displacement and travel time, health macroregion II of PE, 2012.

Usual-risk deliveries				High-risk deliveries			
	
Geographic displacement	Travel time	# of childbirths	% of childbirths	Geographic displacement	Travel time	# of childbirths	% of childbirths
< 30 km	Up to 30 minutes	1,557	21.22	< 30 km	Up to 30 minutes	311	6.30
≥ 30 km and < 60 km	30 - 60 minutes	2,841	38.72	≥ 30 km and < 60 km	30 - 60 minutes	978	19.82
≥ 60 km and < 90 km	30 - 90 minutes	1,329	18.11	≥ 60 km and < 90 km	30 - 90 minutes	1,015	20.57
≥ 90 km and < 120 km	90 - 120 minutes	620	8.45	≥ 90 km and < 120 km	90 - 120 minutes	205	4.16
< 120 km	Above 120 minutes	991	13.50	< 120 km	Above 120 minutes	2,425	49.15

Total		7,338	100.00			4,934	100.00

Source: SIH/Datasus, 2018.

**Figure 2:**
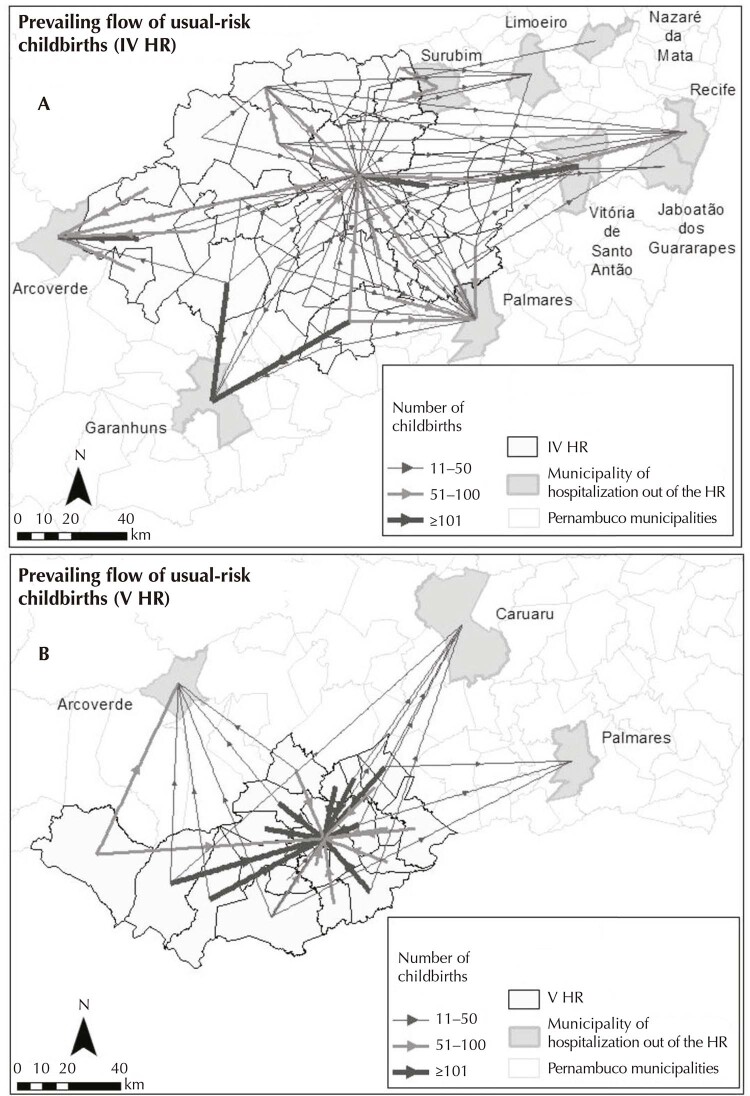
Prevailing flow of displacements of pregnant women with usual-risk deliveries from IV health region (a) and V health region (b), in 2018.

Source: SIH/Datasus, 2018.

Figure 2 shows on the spatial analysis how these distances are covered by the dominant flow of the usual risk pregnant women for delivery in health regions IV (A) and V (B). The municipalities outside the IV region, which performed usual risk childbirths from women residing in the same region, were: Arcoverde, Garanhuns, Palmares, Surubim, Vitória de Santo Antão, Limoeiro, Jaboatão dos Guararapes and Recife; for women in the V region were: Arcoverde, Caruaru and Palmares.

The IV health region managed to perform 75.2% of childbirths from pregnant women at usual risk within its territory, while the V region performed 87.8% of these childbirths. When considering the two largest municipalities in macroregion II, it was found that Caruaru and Garanhuns performed 97% and 97.3% of these childbirths in their territory, respectively. However, for smaller municipalities, almost half of the women had their children outside their municipality of residence.

**Figure 3:**
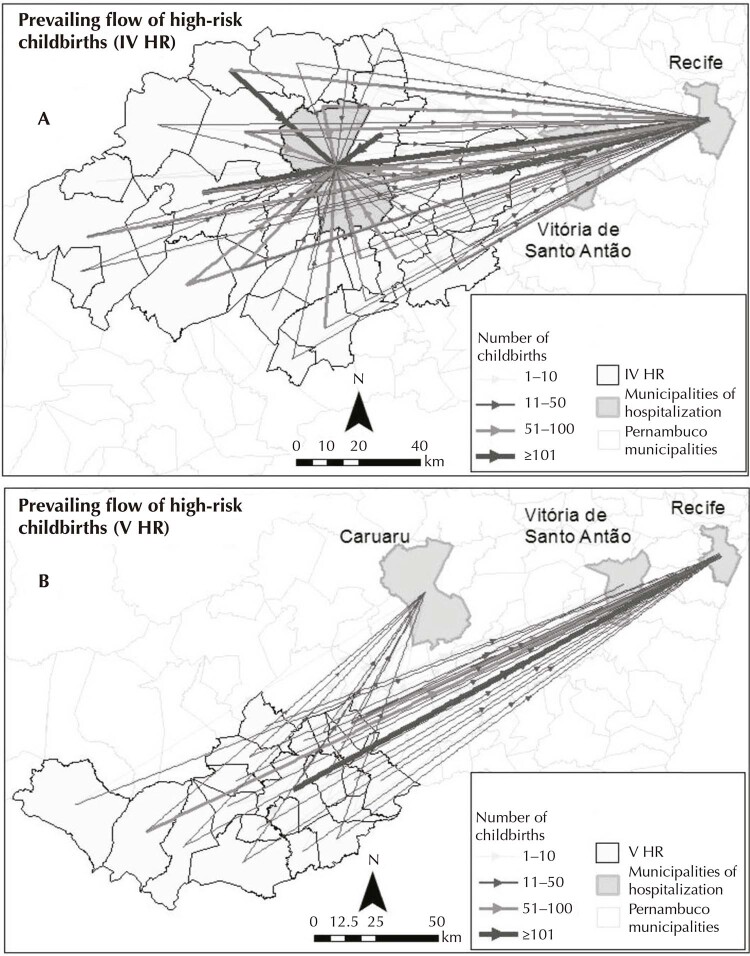
Prevailing flow of displacements of pregnant women with high-risk deliveries from IV health region (a) and V health region (b) in 2018.

Source: SIH/Datasus, 2018.

When analyzed only the childbirths from high-risk pregnant women, the reference maternity hospital of macroregion II performed only 46.9% of deliveries of women residing in this macroregion. Therefore, the other high-risk childbirths were performed in macroregion I, especially in the cities of Recife (89.1%) and Vitória de Santo Antão (10.7%). Of the childbirths from high-risk pregnant women living in the IV region (Figure 3 (A)), 51.7% were performed in macroregion II and the others in macroregion I. Of the total number of deliveries referred, 88.2% were performed in Recife and 11.8% in Vitória de Santo Antão. The municipality of Caruaru, the reference center for high-risk deliveries from pregnant women in macroregion II, referred 52.8% of these deliveries (2,621 deliveries) to macroregion I, of which 92.9% (2,336) were to the municipality of Recife, 7.1% (280) to Vitória de Santo Antão and 0.2% to other municipalities in macroregion I.

Only 26.9% of deliveries from high-risk pregnant women residing in the V region were performed in macroregion II and the remainder was referred to macroregion I, mainly to the cities of Recife and Vitória de Santo Antão (Figure 3 (B)). It is noteworthy that women living in the 5th region can travel up to 335 km to Recife in search for high-risk obstetric care, as is the case of the municipality of Itaíba. The municipality of Águas Belas, 184 km away from the municipality where the macroregion II is located and 315 km away from Recife, had 72.9% of the childbirths from high-risk pregnant women in the municipality of Recife (86 deliveries) and 27.1% in Caruaru (32 deliveries).

**Figure 4:**
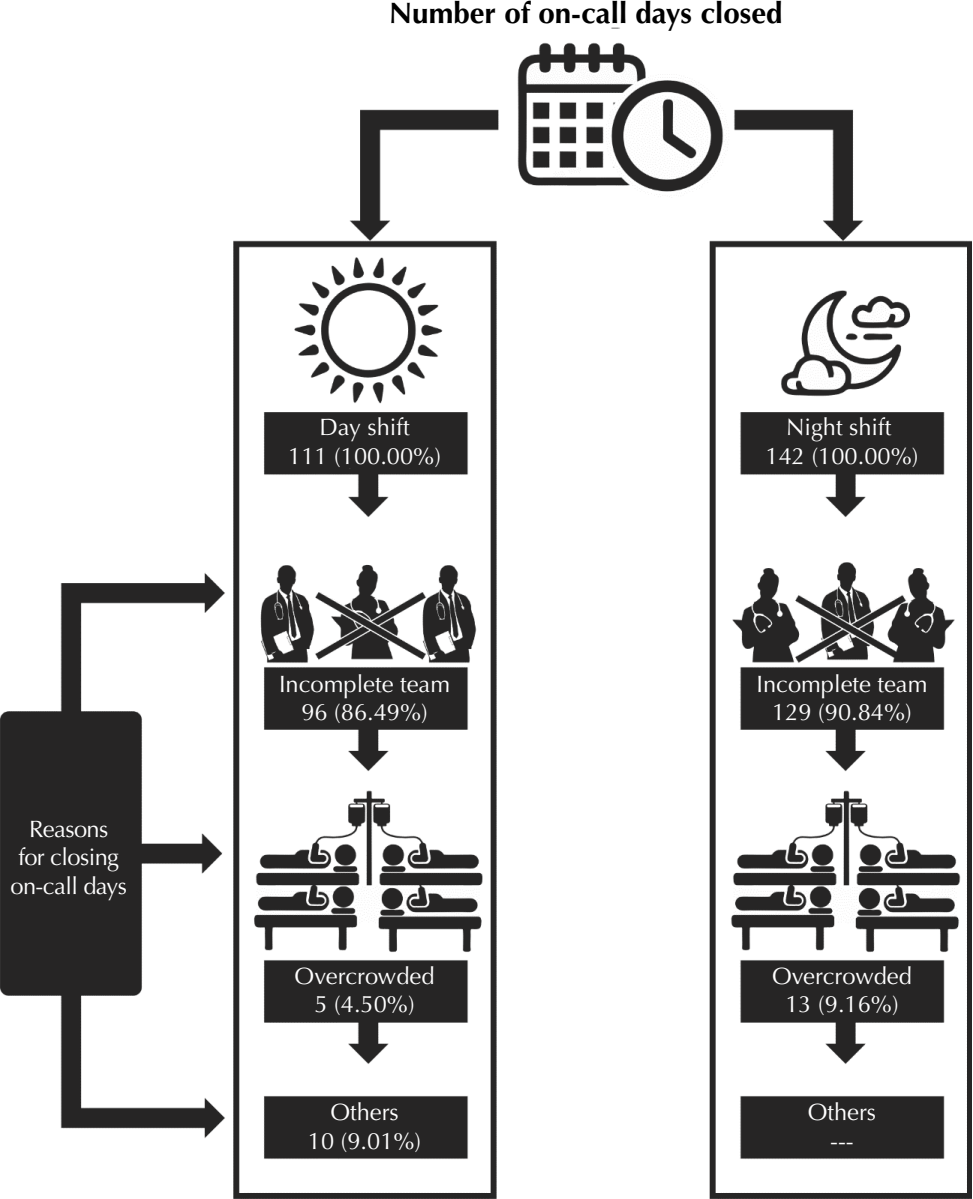
Number of on-call days closed for admission to delivery and the reason for closure, reference maternity hospital of health macroregion II, 2018.

Source: Source: SES/GRH/Regmed, 2018.

Regarding obstetric care to high-risk pregnant women, in 2018 the reference maternity hospital of health macroregion II had 30.4% of day shifts and 38.9% of night shifts closed for admission of pregnant women (Figure 4), and the main reason for closing shifts was the shortage of on-call team.

## DISCUSSION

Determining the risk of a pregnancy is important to develop actions that may reduce maternal and infant morbidity and mortality. In this study, 22.3% of the pregnancies reviewed were considered of high-risk. These findings are close to those found by Viellas et al.^15^ in a national survey, showing that a quarter of pregnant women in the Northeast region were considered high-risk, especially those belonging to the age extremes (under 15 years and women of 35 years or older), with three or more previous pregnancies and those with negative outcomes in previous pregnancies.

The V health region showed lower proportions of high-risk pregnant women. This is possibly due to the inadequate classification of childbirths as to risk, since in this region there is no maternity hospital to serve the high obstetric risk. The reference maternity hospital for this region is located in the IV health region. This fact leads to a false impression that among women residing in the 5th region there is a lower proportion of pregnant women with high obstetric risk when, in fact, what happens is that in the absence of an appropriate high-risk obstetric service, many women have this type of delivery in reference maternity hospitals for usual risk.

The organization of health regions and macroregions are important instances to guarantee access to healthcare services; however, their conformation does not guarantee that access to these services is facilitated. Although the efforts made to organize services on a macro scale are acknowledged, the difficulty in accessing more complex services still remains. Moreover, there are difficulties in organizing local systems^8^.

From the perspective of obstetric care, the *Rede Cegonha* advocates the guarantee by the municipalities of access to care for pregnant women and, if necessary, referral to services of different technological densities^2^. High percentages of pregnant women traveling in search of a place to give birth demonstrate barriers to access. However, researchers have observed in a nationwide study that only 58.7% of pregnant women interviewed received guidance on the link to the maternity unit of reference for childbirth^16^.

One of the alternatives for structuring the *Rede Cegonha* in health macroregions to avoid unnecessary displacement and, consequently, improve access is the implementation of Normal Delivery Centers (*Centros de Parto Normal*, CPN). The CPN aim to contribute to the redefinition of the childbirth care model, rescuing the right to privacy and dignity of women by giving birth in a place close to their family environment, with appropriate technological resources in case of need^17^.

Regarding high-risk pregnant women, many had to travel a long way, more than two hours, to deliver in macroregion I, showing barriers of access to more complex services. This fact has already been pointed out by Almeida et al.^13^, when they observed differences in the supply of childbirth care services among regions and states in Brazil.

In Pernambuco there is a concentration of high risk obstetric care services in macroregion I, mainly in the state capital. In macroregions II and IV there are two high-risk maternity hospitals. However, in macroregion II there is no obstetric Intensive Care Unit (ICU), although there is a neonatal Intermediate Care Unit (IMCU). This scenario reveals the non-incorporation of the concept of access, notably the dimension “availability and accommodation”^12^, reflecting the disorder of the obstetric care network.

This inadequacy has also been documented in research conducted in all regions of Brazil^18^. In Pernambuco, there are only two obstetric ICUs, one located in the capital and the other in the *sertão* region of the state. The late access to services contributes to the worsening of many situations when obstetric complications occur.

The excessive occupation of obstetric beds in macroregion I, due to its concentration of high-risk care services, has as a consequence the displacement of women residing in other municipalities^6^. On the other hand, Silva et al.^19^ found that most users assisted in the healthcare network of Recife lived outside the capital. This finding shows the breakdown of the obstetric care network in Pernambuco, leading to the pilgrimage of women in search of a suitable place for delivery, a fact also observed in another study^20^.

Pilgrimage for childbirth has also been documented in other nationwide studies, where the Northeast presented the worst results (25.1%)^15^, as well as in research conducted in São Luiz and Ribeirão Preto^21^.

Although the existence of a regionalized network presumes displacement between care sites, these referrals should happen in a coordinated manner, through organized flows and well-defined protocols, avoiding pilgrimage and, consequently, expanding the access of women to healthcare services. However, the findings of the current survey revealed the persistence of difficulties in the provision of care to high obstetric risk, especially when one observes a high proportion of closed shifts, mainly due to insufficient professionals. This fact may discredit the service, causing women to seek care in other health units.

In macroregion II, the shortage of professionals, especially physicians, is a recurring problem. This problem has already been observed by Araújo^22^ in 2012. The author verified that, although there were enough obstetric beds, there was a deficit of approximately 40% of obstetricians and more than 50% of anesthesiologists. Several authors have pointed out that the “absence of a doctor on duty” was the main difficulty for women to access care for childbirth in the first health unit searched^6,23^.

The study *Região e Redes* (Region and Networks), conducted in the five Brazilian regions, pointed out the insufficient physical capacity and unavailability of human resources, besides the large gaps in healthcare still found throughout the Brazilian territory, as important points that contribute to hinder the regionalization policy in the country, resulting in disengagement of services^23^.

In this survey, we observed barriers to the access of pregnant women, especially for those with obstetric high-risk deliveries. It is a worrisome fact in the perspective of worsening problems for the access to the obstetric care network, due to the Covid-19 pandemic^24^. The difficulty of access to healthcare services beyond the reach of women in an adequate and timely way is considered a violation of human rights.

The study design presented some limitations. Using the centroid rather than the woman’s address to calculate the distance is a considerable limitation, because the distance to the service in the same municipality may be greater than to reach the service in a neighboring municipality, even from another health macroregion.

Another limitation is that the analysis of access failed in including aspects such as cost and means of transportation used for displacement, and it was not possible to identify the reasons for long trips in search of hospital care for childbirth. This shows that the option to work with one of the five dimensions of access (availability and accommodation) brought limitations to the analysis, since these dimensions are interrelated.

Thus, it is suggested that future studies should bring to the analysis the experience of women who are seeking healthcare services, the aspects of individuals, services and the context in which they are inserted. However, it is indisputable how the availability and accommodation dimension disclosed that many women from the interior of Pernambuco travel long distances in search of obstetric care.

This is not only a local reality, being widely evidenced in other regions of Brazil^25^. Although Brazil is considered to have improved the access of users to good practices and appropriate technologies for childbirth, especially in the Northeast region, with influence of the Stork Network in this process, as shown by Leal et al.^26^, the findings of this research reveal that problems related to the dimension of access regarding availability and accommodation still persist, as evidenced in the macroregion II of Pernambuco. Thus, it is considered that the obstetric healthcare network in the state is not structured to ensure equitable access to birth care, which highlights the need for its restructuring and approximation with the recommendations of the *Rede Cegonha*.
