# Supplementary material for: Access to childbirth care services in the interior of Pernambuco, Northeast region of Brazil
Source: Rev Saude Publica. 2023 Mar 15;57:7. doi: 10.11606/s1518-8787.2023057004335 (PMC10118422; doi:10.11606/s1518-8787.2023057004335)
Supplement: Translation

# Translation: Acesso aos serviços de atenção ao parto no interior de Pernambuco, Nordeste do Brasil

**Authors:** Régia Maria Batista Leite, Thália Velho Barreto de Araújo, Maria Rejane Ferreira da Silva, Antônio da Cruz Gouveia Mendes, Maria do Socorro Veloso de Albuquerque

**Affiliations:** I Brasil Fundação Oswaldo Cruz. Instituto Aggeu Magalhães. Programa de Pós-Graduação em Saúde Pública. Recife, PE, Brasil; II Brasil Universidade Federal de Pernambuco. Centro de Ciências Médicas. Programa de Pós-Graduação em Saúde Coletiva. Recife, PE, Brasil; III Brasil Universidade de Pernambuco. Faculdade de Enfermagem Nossa Senhora das Graças. Recife, PE, Brasil; IV Brasil Fundação Oswaldo Cruz. Instituto Aggeu Magalhães. Departamento de Saúde Pública. Recife, PE, Brasil

**Keywords:** Serviços de Saúde Materno-Infantil, provisão & distribuição, Qualidade, Acesso e Avaliação da Assistência à Saúde, Barreiras ao Acesso aos Cuidados de Saúde, Estudos Ecológicos

## Abstract

**OBJETIVO:**

Analisar o acesso de mulheres atendidas na rede pública aos serviços de atenção ao parto, destacando-se as barreiras relacionadas à dimensão “disponibilidade e acomodação” em uma macrorregião de saúde de Pernambuco.

**MÉTODOS:**

Estudo ecológico, realizado a partir dos registros de partos hospitalares do Sistema de Informação Hospitalar e de informações da Central de Regulação de Leitos do estado sobre mulheres residentes na macrorregião de saúde II, em 2018. Analisou-se os deslocamentos, considerando a distância geográfica entre o município de residência e o de ocorrência do parto, o tempo estimado do deslocamento das gestantes, a proporção de plantões bloqueados para admissão das gestantes para o parto e o motivo da indisponibilidade.

**RESULTADOS:**

Em 2018, a macrorregião de saúde II realizou 84% dos partos de risco habitual e 46,9% de alto risco. Os demais partos de alto risco (51,1%) ocorreram na macrorregião I, sobretudo no Recife. A maternidade de referência para partos de alto risco dessa macrorregião teve 30,4% dos dias de plantões diurnos bloqueados para admissão de partos e 38,9% dos noturnos; o principal motivo foi a dificuldade em manter a equipe completa no serviço.

**CONCLUSÕES:**

Mulheres residentes na macrorregião de saúde II de Pernambuco enfrentam grandes barreiras de acesso em busca de atendimento hospitalar para o parto, percorrendo grandes distâncias, mesmo quando gestantes de risco habitual, levando à peregrinação em busca dessa assistência. Há dificuldade de disponibilidade e acomodação nos serviços de alto risco e de emergências obstétricas, com insuficiente capacidade física e de recursos humanos. A rede de atenção obstétrica na macrorregião II de Pernambuco não está estruturada para garantir um acesso equânime à assistência das gestantes no momento do parto, o que evidencia a necessidade de sua reestruturação em aproximação ao preconizado pela Rede Cegonha.

## INTRODUÇÃO

Nas três últimas décadas, o Brasil vem apresentando avanços na atenção à saúde das mulheres, em decorrência de esforços e iniciativas do governo e da sociedade, em especial do movimento organizado de mulheres. No entanto, ainda persistem elevados níveis de morbimortalidade materna, fetal e neonatal evitáveis^1^.

Essa persistente situação justificou a implantação de vários programas, políticas e estratégias de saúde, entre elas a Rede Cegonha (RC), lançada em 2011, como estratégia para organização da assistência materna e infantil, com o objetivo de mudança do modelo de atenção ao parto e nascimento e de redução da morbimortalidade materna e neonatal^2^.

Essa mudança de modelo da atenção obstétrica e neonatal foi discutida por gestores nas diversas regiões de saúde do país, inclusive com a adesão da macrorregião de saúde II de Pernambuco^3^, firmando-se compromissos de alteração das práticas de atenção ao parto e nascimento^4,5^. Essa organização da rede é importante para assegurar o acesso das mulheres aos serviços de saúde, em especial aos de atenção ao parto, evitando a ocorrência de fluxos desordenados de gestantes, que podem levar à peregrinação das mulheres em busca de atendimento para o parto^6^. Um aspecto importante para superar as dificuldades de acesso é a organização dessa rede para que vença a persistência de grandes diversidades na distribuição e na qualidade dos serviços ofertados, entre regiões e macrorregiões de saúde^7^.

No entanto, sabe-se que apesar dos diversos esforços para prover ações e serviços nas regiões de saúde, concorda-se com Shimizu et al.^8^ quando dizem que o acesso aos serviços de saúde, sobretudo os de maior complexidade, aos serviços especializados e a hospitais com mais recursos tecnológicos, é difícil, exigindo que os usuários percorram grandes distâncias para conseguirem o atendimento necessário.

Nesse sentido, entende-se que o acesso aos serviços de saúde é um componente importante em todos os sistemas de saúde. Entretanto, não há consenso na literatura sobre o seu conceito^9,10^, variando entre autores e mudando ao longo do tempo^11^. Para efeito do presente artigo, adotou-se o conceito de acesso sistematizado por Levesque et al.^12^, em que é definido como a oportunidade de alcançar e obter serviços de saúde adequados em situações de necessidade percebida de cuidados, sendo resultado da interface entre as características de pessoas, famílias, ambientes sociais e físicos e as características dos sistemas, organizações e prestadores de saúde.

Em seu modelo teórico os autores formularam cinco dimensões que envolvem o conceito de acesso (acessibilidade, aceitabilidade, disponibilidade e acomodação, acessibilidade financeira e adequação) e cinco respectivas capacidades individuais dos usuários (capacidade de perceber; de procurar; de alcançar; de pagamento; de se envolver). Neste artigo optou-se por trabalhar com a dimensão “disponibilidade e acomodação”, que abarca a existência física e o potencial de produção do serviço e envolve os aspectos estruturais, as instalações, a distribuição, os recursos humanos, os contextos e a geografia urbana. Configura-se no fato de alcançar um serviço de saúde e em tempo oportuno^12^.

Nessa direção, considerou-se importante analisar o acesso de mulheres atendidas na rede pública aos serviços de atenção ao parto na macrorregião de saúde II de Pernambuco, destacando-se as barreiras relacionadas à dimensão “disponibilidade e acomodação”.

## MÉTODOS

Foi realizado um estudo ecológico de base populacional a partir dos registros de partos hospitalares do Sistema de Informação Hospitalar (SIH) e de informações da Central de Regulação de Leitos do Estado de Pernambuco sobre mulheres residentes na macrorregião de saúde II do estado, no período entre primeiro de janeiro e 31 de dezembro de 2018.

Em Pernambuco, a organização da rede está normatizada no Plano Diretor de Regionalização (PDR), com a redefinição da organização territorial do estado em 12 regiões de saúde agrupadas em quatro macrorregiões de saúde, com o objetivo de subsidiar a organização da rede de saúde de forma regionalizada, hierárquica e resolutiva^3^. A macrorregião de saúde II está localizada no Agreste do Estado de Pernambuco e é composta pelas regiões de saúde IV e V. A IV região de saúde possui sede administrativa em Caruaru e compreende 32 municípios, com uma população de 1.324.382 habitantes^3^ (415.471 mulheres em idade fértil) e dispõe de uma maternidade que é a referência da macrorregião II para gestantes de alto risco, mas sem UTI para essas gestantes. A V região de saúde, com sede administrativa no município de Garanhuns, é composta por 21 municípios e 534.793 habitantes^3^ (163.667 mulheres em idade fértil) e possui uma maternidade regional para atendimento de gestantes de risco habitual.

Para analisar o acesso por meio da dimensão “disponibilidade e acomodação”, proposta por Levesque et al.^12^, foram utilizados os deslocamentos das mulheres por meio das variáveis: distância percorrida pelas mulheres em busca de assistência ao parto de risco habitual e de alto risco obstétrico e o tempo de deslocamento entre o município de residência e o de ocorrência. Também foram analisadas variáveis relacionadas ao serviço de atendimento a partos de alto risco obstétrico, referência para as mulheres da macrorregião II, para isso foram utilizadas as seguintes variáveis: proporção de dias de plantões diurnos e noturnos fechados para admissão de gestantes no momento do parto e o motivo do fechamento dos plantões, a partir de informações da Central de Regulação de Leitos de Pernambuco, que funciona com o apoio de um *call center*, que recebe ligações de todas as unidades de saúde do Estado que lidam com casos de urgência/emergência, UTI e obstetrícia. A central consolida as informações sobre as demandas e os atendimentos realizados.

As distâncias intermunicipais foram geoprocessadas a partir de cálculos das distâncias entre os centroides dos municípios de residência e ocorrência. No cálculo do deslocamento geográfico, não foram considerados os deslocamentos intramunicipais; portanto, se o parto ocorreu no próprio município de residência, a distância foi igual a zero, segundo metodologia proposta por Almeida et al^13^.

As distâncias geográficas e o tempo de deslocamento foram calculados estimados e agrupados em cinco categorias (< 30km/30min.; ≥ 30 - < 60km/30 a 60min.; ≥ 60 - < 90km; ≥ 90km < 120km/90 a 120min.; e < 120km/acima de 120 min.), incluindo todos os municípios da macrorregião II, levando em consideração as gestantes na ocasião por partos de risco habitual e de alto risco, de acordo as definições do Ministério da Saúde, incorporadas pelo SIH. O banco do SIH e as informações sobre o funcionamento dos plantões foram cedidos pela Secretaria Estadual de Saúde de Pernambuco.

Para a análise dos deslocamentos das gestantes foram construídos mapas de fluxo por meio do programa ArcGIS 10.4, diferenciados para gestantes com partos de risco habitual e para gestantes com partos de alto risco, tendo como unidade de análise as regiões de saúde e a macrorregião de saúde.

Os mapas de fluxo representam o deslocamento das mulheres no espaço, demonstrando o movimento por meio de vetores traçados para representar o itinerário percorrido pelas gestantes, caracterizados por meio de espessuras proporcionais ao número de partos. Os fluxos foram categorizados para as gestantes de partos de risco habitual: IV região de saúde (11–50 partos; 51–100 partos; e ≥ 101 partos) e para a V região de saúde (11–50; 51–100; e ≥ 101 partos). Já para os partos de gestantes de alto risco, os fluxos foram definidos da mesma forma para as duas regiões de saúde: 1–10 partos; 11–50 partos; 51–100 partos; e ≥ 101 partos. Nesse sentido, o fluxo dominante foi definido como o maior fluxo (a partir de 10 partos) de cada município, o que permitiu identificar o arcabouço da rede de ligações^14^.

O estudo dos fluxos é componente fundamental para observação de questões ligadas ao acesso, identificando as distâncias percorridas^14^, ajudando a identificar a organização dos serviços em rede regionalizada. Do mesmo modo, a organização do serviço de atenção ao parto de alto risco obstétrico também se configura um importante componente de “disponibilidade e acomodação” para observar em que medida o serviço está estruturado na macrorregião de saúde para diminuir barreiras de acesso.

A pesquisa foi aprovada pelo Comitê de Ética em Pesquisa do Centro de Pesquisas Aggeu Magalhães/Fiocruz, conforme Resolução 510/16, sob CAAE n. 63796717.4.0000.5190.

## RESULTADOS

Em 2018, 77,7% dos partos ocorridos na macrorregião de saúde II foram classificados como de risco habitual e 22,3% de alto risco. Ao considerar as regiões de saúde, verificou-se que 26,4% dos partos de gestantes de alto risco foram de residentes na IV região de saúde, enquanto na V região esse valor foi de 13,5% (Figura 1).

**Figura 1:** Proporção de partos de risco habitual e alto risco segundo região de saúde de residência e macrorregião de saúde II, 2018.

Fonte: SIH/Datasus, 2018.

No que se refere ao local de realização dos partos, observou-se que 84% dos partos de gestantes de risco habitual foram realizados na própria macrorregião de saúde II. Todavia, ao serem analisados os partos de risco habitual e de alto risco obstétrico ocorridos fora da região de residência, observou-se que 66,4% dos partos de gestantes de risco habitual ocorrem em municípios com distância de até 30 km e, 17,1% ocorrem em municípios com mais 120 km. Com relação aos partos de gestantes de alto risco, 49,1% das mulheres necessitaram se deslocar mais de 120 km, demorando mais de duas horas de viagem para ter o parto ou emergência obstétrica atendida. Esses resultados demonstram que muitas mulheres tiveram que se deslocar para municípios muito distantes para conseguir assistência, mesmo sendo gestantes de risco habitual (Tabela 1).

**Tabela 1:** Distribuição dos partos de risco habitual e de alto risco obstétrico, segundo os deslocamentos geográficos e o tempo de deslocamento, macrorregião de saúde II de PE, 2012.

Partos de risco habitual				Partos de alto risco			
	
Deslocamento geográfico	Tempo de deslocamento	Nº de partos	% de partos	Deslocamento geográfico	Tempo de deslocamento	Nº de partos	% de partos
< 30 km	Até 30 minutos	1.557	21,22	< 30 km	Até 30 minutos	311	6,30
≥ 30 km e < 60 km	De 30 a 60 minutos	2.841	38,72	≥ 30 km e < 60 km	De 30 a 60 minutos	978	19,82
≥ 60 km e < 90 km	De 60 a 90 minutos	1.329	18,11	≥ 60 km e < 90 km	De 60 a 90 minutos	1.015	20,57
≥ 90 km e < 120 km	De 90 a 120 minutos	620	8,45	≥ 90 km e < 120 km	De 90 a 120 minutos	205	4,16
< 120 km	Acima de 120 minutos	991	13,50	< 120 km	Acima de 120 minutos	2.425	49,15

Total		7.338	100,00			4.934	100,00

Fonte: SIH/Datasus, 2018.

Na Figura 2, pode-se observar na análise espacial como essas distâncias são percorridas por meio do fluxo dominante dos deslocamentos das gestantes de risco habitual para realização do parto nas regiões de saúde IV (A) e V (B). Os municípios fora da IV região, que realizaram partos de risco habitual de mulheres residentes na mesma região, foram: Arcoverde, Garanhuns, Palmares, Surubim, Vitória de Santo Antão, Limoeiro, Jaboatão dos Guararapes e Recife; para as mulheres da V região foram: Arcoverde, Caruaru e Palmares.

**Figura 2:** Fluxo dominante dos deslocamentos de gestantes com partos de risco habitual da IV região de saúde (a) e V região de saúde (b), em 2018.

Fonte: SIH/Datasus, 2018.

A IV região de saúde conseguiu realizar 75,2% dos partos de gestantes de risco habitual dentro do seu território, enquanto a V região realizou 87,8% desses partos. Ao considerar os dois maiores municípios da macrorregião II, verificou-se que Caruaru e Garanhuns realizaram 97% e 97,3% desses partos em seu território, respectivamente. Entretanto, para os municípios menores, quase a metade das mulheres tiveram seus filhos fora do seu município de residência.

Quando analisados apenas os partos de gestantes de alto risco, a maternidade de referência da macrorregião II realizou apenas 46,9% dos partos de munícipes residentes nessa macrorregião. Como consequência, os demais partos de gestantes de alto risco foram realizados na macrorregião I, sobretudo nos municípios de Recife (89,1%) e Vitória de Santo Antão (10,7%). Dos partos de gestantes de alto risco de mulheres residentes na IV região (Figura 3 (A)), 51,7% foram realizados na própria macrorregião II e os demais na macrorregião I. Do total de partos encaminhados, 88,2% foram realizados no Recife e 11,8% em Vitória de Santo Antão. O município de Caruaru, sede da maternidade de referência para partos de gestantes de alto risco da macrorregião II, encaminhou 52,8% desses partos (2.621 partos) para a macrorregião I, dos quais 92,9% (2.336) foram para o município de Recife, 7,1% (280) para Vitória de Santo Antão e 0,2% para outros municípios da macrorregião I.

**Figura 3:** Fluxo dominante dos deslocamentos de gestantes com partos de alto risco da IV região de saúde (a) e da V região de saúde (b) em 2018.

Fonte: SIH/Datasus, 2018.

Dos partos de gestantes de alto risco de residentes na V região apenas 26,9% foram realizados na macrorregião II e o restante fora encaminhado para macrorregião I, principalmente para os municípios de Recife e Vitória de Santo Antão (Figura 3 (B)). Ressalta-se que mulheres residentes na V região podem percorrer uma distância de até 335 km para Recife em busca de assistência obstétrica de alto risco, como é o caso do município de Itaíba. O município de Águas Belas, distante 184 km do município sede da macrorregião II e 315 km de distância do Recife, teve 72,9% dos partos de gestantes de alto risco realizados no município do Recife (86 partos) e 27,1% em Caruaru (32 partos).

Com relação a assistência obstétrica de gestantes de alto risco, em 2018, a maternidade de referência da macrorregião de saúde II, teve 30,4% dos plantões diurnos e 38,9% dos noturnos fechados para admissão de gestantes (Figura 4) e o principal motivo para fechamento dos plantões foi a incompletude da equipe de plantão.

**Figura 4:** Número de dias de plantão fechado para admissão ao parto e o motivo do fechamento, maternidade de referência da macrorregião de saúde II, 2018.

Fonte: Fonte: SES/GRH/Regmed, 2018.

## DISCUSSÃO

A determinação do risco de uma gestação é importante para se desenvolver ações que possam reduzir a morbimortalidade materna e infantil. Neste estudo, foram consideradas de alto risco 22,3% das gestações analisadas. Esses achados são próximos daqueles encontrados por Viellas et al.^15^, em inquérito de base nacional, evidenciando que um quarto das gestantes da região Nordeste eram considerados alto de risco, principalmente aquelas pertencentes aos extremos etários (menores de 15 anos e mulheres com 35 anos ou mais de idade), com três ou mais gestações anteriores e aquelas com desfechos negativos em gestações anteriores.

A V região de saúde apresentou menores proporções de gestantes de alto risco, o que possivelmente se deve à classificação inadequada dos partos quanto ao risco, já que nessa região não existe maternidade para atender ao alto risco obstétrico. A maternidade de referência para essa região está localizada na IV região de saúde. Esse fato leva a uma falsa impressão de que entre as mulheres residentes na V região há menor proporção de gestantes de alto risco obstétrico, quando, na verdade, o que ocorre é que, na ausência de um serviço obstétrico de alto risco adequado, muitas mulheres realizam esse tipo de parto em maternidades de referência para risco habitual.

A organização das regiões de saúde e macrorregiões são instâncias importantes para garantia de acesso aos serviços de saúde, entretanto sua conformação não garante que o acesso a esses serviços seja facilitado. Embora se reconheça os esforços realizados para organização dos serviços em escala macro, ainda permanece a dificuldade de acesso aos serviços de maior complexidade. Ademais, há dificuldades na organização dos sistemas locais^8^.

Na perspectiva da atenção obstétrica, a Rede Cegonha preconiza a garantia, por parte dos municípios, de acesso ao atendimento das gestantes e, se necessário, o encaminhamento a serviços de diferentes densidades tecnológicas^2^. Altos percentuais de deslocamentos das gestantes em busca de um local para o parto demonstram barreiras de acesso. Um importante componente da rede para superação das barreiras de acesso é a vinculação da gestante ao local do parto, entretanto, pesquisadores já observaram, em estudo de abrangência nacional, que somente 58,7% das gestantes entrevistadas receberam orientação sobre à vinculação à maternidade de referência para o parto^16^.

Uma das alternativas para estruturação da Rede Cegonha nas macrorregiões de saúde para evitar deslocamentos desnecessários e, consequentemente, melhorar o acesso é a implantação dos Centros de Parto Normal (CPN), que têm como objetivo contribuir para a redefinição do modelo assistencial ao parto, resgatando o direito à privacidade e à dignidade da mulher ao dar à luz num local próximo ao seu ambiente familiar, com recursos tecnológicos apropriados em casos de eventual necessidade^17^.

Com relação às gestantes de alto risco, muitas mulheres tiveram que realizar grandes deslocamentos, com mais de duas horas de viagem para realização do parto na macrorregião I, evidenciando barreiras de acesso para serviços de maior complexidade. Esse fato já foi apontado por Almeida et al.^13^, quando observou diferenças na oferta dos serviços de atenção ao parto entre as regiões e estados do Brasil.

Em Pernambuco há uma concentração de serviços de atenção ao alto risco obstétrico na macrorregião I, principalmente na capital do estado. Nas Macrorregiões II e IV existem duas maternidades de alto risco. Porém, na macrorregião II não existe Unidade de Terapia Intensiva (UTI) obstétrica, embora tenha Unidade de Cuidados Intermediários (UCI) neonatal. Esse cenário revela a não incorporação do conceito de acesso, em especial da dimensão “disponibilidade e acomodação”^12^, refletindo na desorganização da rede de assistência obstétrica.

Essa inadequação também tem sido documentada em pesquisa realizada em todas as regiões do Brasil^18^. Em Pernambuco só existem duas UTIs obstétricas, uma situada na capital e outra no sertão do estado. A demora no acesso ao serviço contribui para o agravamento de muitas situações quando da ocorrência de complicações obstétricas.

A excessiva ocupação de leitos obstétricos na macrorregião I, em razão de sua concentração de serviços de atenção ao alto risco, tem como consequência o deslocamento das mulheres residentes para outros municípios^6^. Por outro lado, Silva et al.^19^ identificaram que a maioria das usuárias atendidas na rede de saúde do Recife residiam fora da capital, evidenciando a desestruturação da rede de atenção obstétrica em Pernambuco, levando à peregrinação das mulheres em busca de um local adequado para o parto, fato também já observado em outro estudo^20^.

A peregrinação por ocasião do parto também foi documentada em outros estudos de abrangência nacional, onde o Nordeste apresentou os piores resultados (25,1%)^15^, bem como em pesquisa realizada em São Luiz e Ribeirão Preto^21^.

Embora a existência de rede regionalizada pressuponha deslocamentos entre os pontos de atenção, essas transferências devem acontecer de forma coordenada, mediante fluxos organizados e por meio de protocolos bem definidos, evitando a peregrinação e, consequentemente, ampliando o acesso das mulheres aos serviços de saúde. Todavia, os achados da presente pesquisa revelaram a persistência de dificuldades na oferta de atenção ao alto risco obstétrico, sobretudo quando se observa uma elevada proporção de plantões fechados, principalmente em razão da insuficiência de profissionais. Esse fato pode desacreditar o serviço, fazendo com que as mulheres busquem atendimento em outras unidades de saúde.

Na macrorregião II a falta de profissionais, sobretudo médicos, é um problema recorrente, já constatado por Araújo^22^, em 2012, que verificou, embora existissem leitos obstétricos em número suficiente, que havia um déficit de aproximadamente 40% de obstetras e de mais de 50% de anestesistas. Diversos autores têm apontado que a “ausência de médico plantonista” foi a principal dificuldade de acesso das mulheres em conseguir atendimento para realização do parto na primeira unidade de saúde procurada^6,23^.

A pesquisa Região e Redes, realizada nas cinco regiões brasileiras, apontou a insuficiente capacidade física e indisponibilidade de recursos humanos, além dos grandes vazios assistenciais ainda presentes em todo território brasileiro, como pontos importantes que contribuem para dificultar a política de regionalização no país, tendo como consequência a desarticulação dos serviços^23^.

Nesta pesquisa, observou-se barreiras de acesso das gestantes, principalmente para aquelas com partos de alto risco obstétrico, fato preocupante na perspectiva do agravamento dos problemas para o acesso à rede de atenção obstétrica, em razão da pandemia da covid-19^24^. A dificuldade de acesso aos serviços de saúde fora do alcance das mulheres em tempo adequado e oportuno é considerada uma violação dos direitos humanos.

O desenho do estudo apresentou algumas limitações. Usar o centroide e não o endereço da mulher para calcular a distância é uma limitação considerável, pois a distância para o serviço do mesmo município pode ser maior do que para chegar ao serviço de um município vizinho, inclusive de outra macrorregião de saúde.

Outra limitação é que a análise do acesso não contemplou aspectos como custo e meios de transporte utilizados para os deslocamentos, não sendo possível identificar os motivos da realização de longos percursos em busca de atendimento hospitalar para o parto. Isso revela que a opção por trabalhar com uma das cinco dimensões do acesso (disponibilidade e acomodação) trouxe limitações à análise, já que essas dimensões são interrelacionadas.

Sendo assim, sugere-se que estudos futuros possam trazer para a análise a experiência das mulheres que estão na busca por serviços saúde, os aspectos dos indivíduos, dos serviços e do contexto em que estão inseridos. No entanto, é indiscutível como, a partir da dimensão disponibilidade e acomodação, foi possível observar que muitas mulheres do interior de Pernambuco percorrem longas distâncias em busca de assistência obstétrica.

Essa não é apenas uma realidade local, sendo amplamente evidenciada em outras regiões do Brasil^25^. Ademais, embora considere-se que no Brasil tenha tido uma melhoria no acesso de usuárias às boas práticas e às tecnologias apropriadas ao parto, sobretudo na região Nordeste, com influência da Rede Cegonha nesse processo, conforme demonstram Leal et al.^26^, os achados da presente pesquisa revelam que ainda persistem problemas relacionados à dimensão de acesso no que se refere a disponibilidade e acomodação como evidenciado na Macrorregional II de Pernambuco. Assim, considera-se que a rede de atenção obstétrica no estado não está estruturada para garantir um acesso equânime à assistência ao parto, o que evidencia a necessidade de sua reestruturação e aproximação com o preconizado pela Rede Cegonha.
